# How did the covid-19 pandemic affect lower respiratory tract infections in young children in England?

**DOI:** 10.1093/eurpub/ckac129.051

**Published:** 2022-10-25

**Authors:** KA Foley, EJ Maile, A Bottle, FK Neale, RM Viner, SE Kenny, A Majeed, DS Hargreaves, S Saxena

**Affiliations:** 1 Department of Primary Care and Public Health, Imperial College London, London, UK; 2 Population, Policy & Practice Department, UCL Great Ormand Street Institute of Child Health, London, UK; 3 Department of Women’s and Children’s Health, Alder Hey Children’s NHS Foundation Trust, Liverpool, UK; 4 NHS England and NHS Improvement, NHS, London, UK; 5 Mohn Centre for Children’s Health, Imperial College London, London, UK

## Abstract

**Background:**

Social distancing policies to reduce transmission of covid-19 also reduced children's exposures to endemic respiratory viruses. We aimed to examine the impact of the covid-19 pandemic on lower respiratory tract infections in under 5s presenting to primary care in England.

**Methods:**

Longitudinal trends analysis using electronic health records from a nationally representative primary care database. Our target population was children aged <5 years registered with a primary care practice from January 2015 to March 2021.

Our main outcome was total weekly contacts with primary care for a lower respiratory tract infection (LRTI). We defined three pandemic phases from March 2020 - March 2021: i) first national lockdown (late March to early June 2020), ii) childcare settings reopened and second national lockdown with schools open (mid-June to mid-December 2020) and iii) third national lockdown with schools closed (late December 2020 to end of March 2021). We compared outcomes during each of the three phases with corresponding calendar weeks during pre-pandemic years 2015 to 2019.

**Results:**

Our study population included 843 020 children <5 years who had 1 076 181 contacts with primary care for LRTIs. During the first phase (first lockdown) there were falls of 79.3% (95% CI: 73.6 to 84.5) from an average of 28 547 primary care contacts for LRTI in 2015 - 2019 to 5915 in 2020; there was a 78.9% (95% CI: 73.7 to 83.9) fall in phase two (childcare settings reopened and second lockdown) from 107 873 to 22 792 contacts; and a 77.7% (95% CI: 73.5 to 81.4) fall in phase three (third lockdown) from 57 200 to 12 764 contacts.

**Conclusions:**

Children under 5 in England had fewer contacts with primary care for LRTIs during the covid-19 pandemic. This change likely reflects lower prevalence of respiratory illness due to fewer social contacts. This may impact on future health service use as these children have had less exposure, and therefore may have less immunity, to respiratory diseases.

**Key messages:**

• Children under 5 had fewer contacts with primary care for lower respiratory tract infections during the covid-19 pandemic in England likely due to the restrictions in place to reduce social contacts.

• The falls in lower respiratory tract infections during the covid-19 pandemic in under 5s may mean they have less immunity to respiratory viruses which may impact upon their future health service use.

